# Supplementary material for: The interactome of intact mitochondria by cross-linking mass spectrometry provides evidence for coexisting respiratory supercomplexes
Source: Mol Cell Proteomics. 2017 Dec 8;17(2):216–32. doi: 10.1074/mcp.RA117.000470 (PMC5795388; doi:10.1074/mcp.RA117.000470)
Supplement: Supplemental Data [file supp_RA117.000470_133922_0_supp_23978_fzffwf.zip › spectra_annotation/mito_spectra_annotation/351-1-1-1-5-1.pdf]

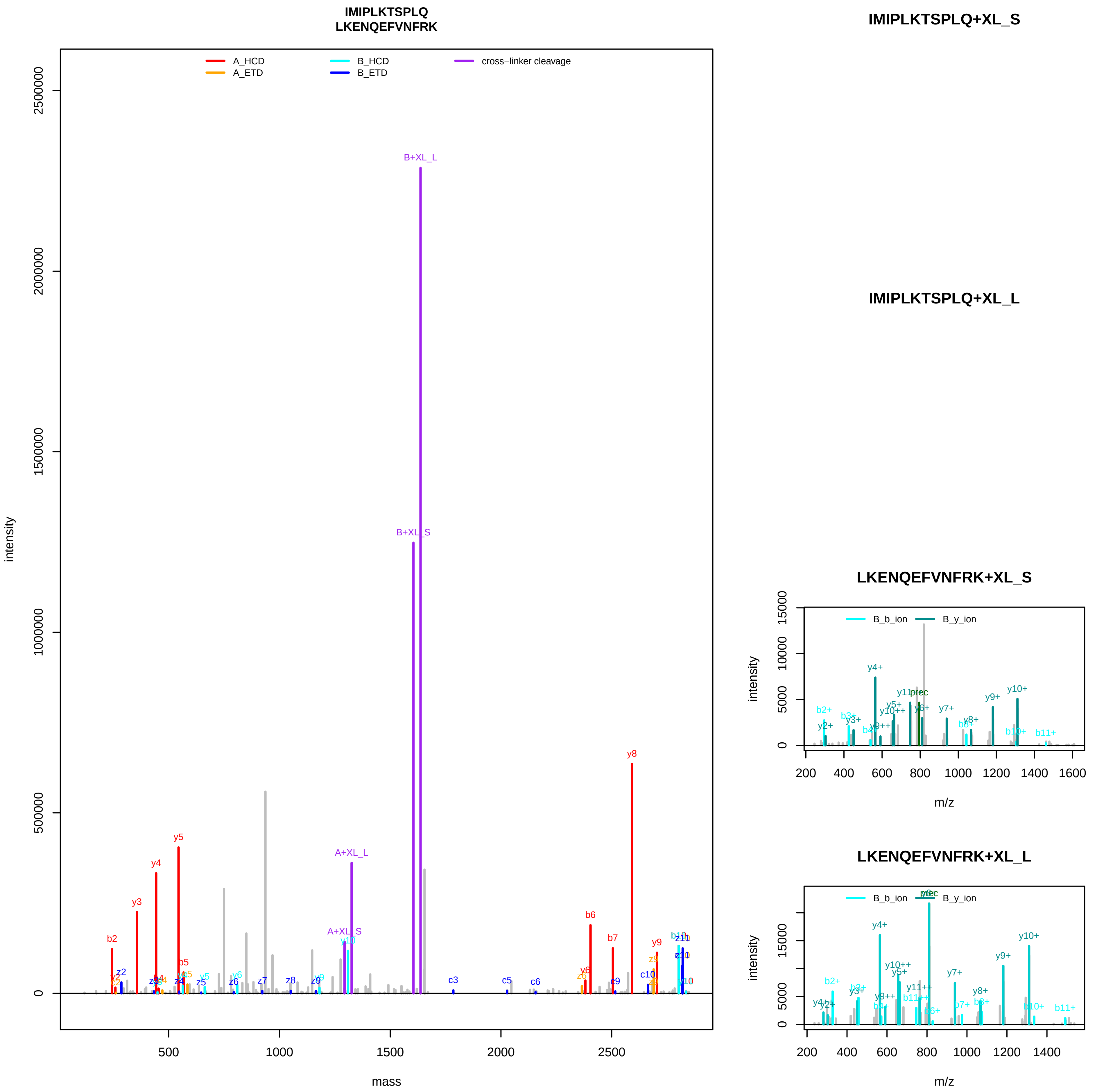
IMIPLKTSPLQ
LKENQEFVNFRK
A_HCD
A_ETD
B_HCD
B_ETD
cross−linker cleavage
B+XL_L
B+XL_S
A+XL_L
A+XL_S
b2
y3
y4
y5
b5
b6
b7
y6
y8
y9
z2
z4
z5
z6
z7
z8
z9
c3
c5
c6
c9
c10
z11
y10
b10
intensity
mass
IMIPLKTSPLQ+XL_S
IMIPLKTSPLQ+XL_L
LKENQEFVNFRK+XL_S
LKENQEFVNFRK+XL_L
B_b_ion
B_y_ion
prec
m/z
